# Multi-scale structural evolution during simulated gelatinization process of sweet potato starch by heat-moisture treatment

**DOI:** 10.1016/j.fochx.2024.102123

**Published:** 2024-12-24

**Authors:** Sijie Zhang, Weiguo Wu, Jihe Zhu, Junling Wu, Zengpeng Gan, Houqin Deng, Yu Zhang, Luyan Liao

**Affiliations:** a College of Food Science and Technology, Hunan Agricultural University, Changsha 410128, China; b Hunan Province Key Laboratory of Food Science and Biotechnology, Changsha 410128, China; c School of Food and Pharmacy, Shanghai Zhongqiao Vocational and Technical University, Shanghai 201514, China

**Keywords:** Heat-moisture treatment, Sweet potato starch, Gelatinization stage, Structure changes

## Abstract

The changes in properties and structures of raw sweet potato starch (RAW-SPS) and heat-moisture treatment (HMT) sweet potato starch (HMT-SPS) during gelatinization process (S1-S6) was investigated to elucidate the improvement effect of HMT on SPS. It was found that SPS exhibited the characteristics of pseudoplastic fluids, characterized by shear thinning and thixotropy, belonged to the C-type starch crystal. The gelatinization temperature of SPS was increased to 82.55 °C by HMT. HMT-SPS had better viscoelasticity in S3-S6. The particle size was smaller than that of RAW-SPS during the gelatinization process. The starch paste of S2 was gelatinized by heating at 95 °C, and the original crystalline structure of the starch was destroyed, where layered structure formed by recrystallization was observed. HMT-SPS showed a clear polarization cross, while disappeared after S2. HMT also improved the ordering degree. The results explained the mechanism of changes in HMT modified starch during the gelatinization process.

## Highlights

• Heat-moisture treatment enhanced the elasticity of sweet potato starch gel.
• Processed sweet potato starch had higher crystallinity and degree of ordering.
• Sweet potato starch exhibited pseudoplastic fluid characteristics and shear thinning.
• Structural changes explained the impact mechanism of heat-moisture treatment.

## 1 Introduction

Sweet potato is widely cultivated, high yield and rich in various nutrients such as starch, protein, vitamins, minerals, etc. Sweet potatoes have edible roots, stems, and leaves, with different nutritional components (Gao et al., 2021). Sweet potato is an important starch crop, widely used in sweet potato vermicelli, noodles, and crisps (Vithu et al., 2019). Sweet potato starch (SPS) is an important source of starch in the food industry, SPS has a higher content of resistant starch, and its glycemic index is lower than other starch foods (Bodjrenou et al., 2023). SPS exhibit low viscosity and poor freeze-thaw stability in food products (Guo et al., 2019), which has great development and utilization prospects. Therefore, it is necessary to modify SPS to enhance its application value, improve the quality and properties of SPS food.

Starch modification technology include physical modification, chemical modification, and enzymatic modification (Compart et al., 2023). Heat moisture treatment (HMT) as an important physical modification method, has the advantages of environmental friendliness and strong adaptability. HMT changed the physicochemical, structure and digestive properties of starch by adjusting the moisture content of starch and treating it for 15–360 min above the gelatinization temperature. HMT is to convert thermal energy into kinetic energy of starch molecules, enhance the movement of molecular chains, accelerate the migration of water molecules between the lamellar, crystalline, and amorphous regions of starch granules, as well as generate new hydrogen bonds, which eventually lead to changes in starch aggregation and chain structure. Several studies proved that HMT significantly affected the physiochemical, structure and digestive properties of potato starch, corn starch, rice starch, sorghum starch, etc. (Chatpapamon et al., 2019). 4–6 h of HMT enhanced the interaction between starch molecules, making the double helix structure more ordered and compact, which improving the viscoelasticity and digestive resistance of modified starch. Dual HMT increased the peak gelatinization temperature by forming resistant starch. Other modification methods combined with HMT were used to improve the properties of starch in a shorter period of time (Fonseca et al., 2021). HMT combined with microwave pretreatment was used to modify potato starch, resulting in a higher gelatinization temperature and significantly increasing the content of slow digestible starch and resistant starch (Deng et al., 2022). As an economical and efficient modified starch technology, many studies had focused on the impact of changing the conditions of HMT on starch. There are few studies on modifying starch based on improving the quality of starch-based products.

Most starch-based foods require heating, and heating gelatinization cause changes in the structure and function of starch. Understanding these changes is important for regulating the quality and nutritional value of starch-based foods. In the gelatinization process, the destruction of the double helix structure in the branched starch structure of corn starch had a significant impact on the multi-scale structure of corn starch. Starch with high amylose content had stronger anti-gelatinization ability, promoted the formation of amylose lipid complexes during gelatinization, and maintained its molecular structure during heating (Zhao et al., 2022). In addition, proteins also interacted with gelatinized starch to form starch lipid protein complexes (Wang et al., 2020). The moisture content and heating temperature are important factors affecting the gelatinization process, the viscosity of gelatinization decreased under low moisture conditions, while under high moisture conditions it increased, which was attributed to the gradual destruction of starch particle structure, followed by the interaction between starch, protein, and lipids (Guo et al., 2018). The gelatinization conditions also affected the rheological properties of starch-based foods, which attributed to the degradation of starch polymers and reduced elastic behavior of flour dispersions. There are few studies comparing and exploring the structural changes during the gelatinization process of SPS. Therefore, it is necessary to clarify the multi-scale structural changes of SPS during the gelatinization process and the mechanism of improving the quality of SPS products, and also provide a new way for the development of starch-based products.

Based on our previous study (Gan et al., 2022), the edible quality and digestive performance of vermicelli made from SPS through HMT were improved, and obtained the optimal HMT conditions. To further investigate the mechanism of HMT in improving the quality of vermicelli made from SPS and its effect on the gelatinization process (divided into six stages based on gelatinization temperature), we comprehensively compared HMT sweet potato starch (HMT-SPS) and raw sweet potato starch (RAW-SPS), used the heating and cooling cycle mode of rapid viscosity analyzer (RVA) to standardize the gelatinization process of SPS and obtain samples in six stages. By analyzing the rheological properties, particle morphology, and crystal structure of SPS, which revealed the mechanism of HMT on SPS properties and the evolution process during gelatinization. This study further expands the practical application of modified SPS in food processing to provide theoretical basis, promotes the utilization and development of sweet potato resources, as well as provides new ideas for the innovative development of HMT technology and SPS.

## 2 Materials and methods

### 2.1 Material

SPS was obtained from Zhangjiajie Cloud Supply and Marketing Co. Ltd. (Hunan, China) and passed through a 150 μm sieve. The total starch, moisture content, apparent amylose, protein of the SPS was 86.29 %, 12.21 %, 30.06 %, and 1.46 %, respectively. Other reagents in this study were of analytical grade.

### 2.2 Preparation of HMT-SPS

The method of Liao et al. (2019) was employed for HMT with slight modification. The starch moisture content was equilibrated to 26 %. The sealed samples (in high-temperature cooking bag) were heated in a hot air oven at 95 °C for 1 h, the starch was freeze-dried and passed through a 100-mesh sieve, the HMT-SPS was obtained for later use.

### 2.3 Sampling during gelatinization stages

Six stages of the gelatinization process samples were prepared using a rapid viscosity analyzer (RVA). The gelatinization program referred to previous research methods (Liao et al., 2021). Sampling at 6 time points during the gelatinization process. The sample was taken when the temperature rose to 80 °C (S1); when the temperature rose 95 °C (S2); after maintaining at 95 °C for 30 min (S3); when the temperature dropped to 50 °C (S4); after maintaining at 50 °C for 30 min (S5); after the temperature dropped to 30 °C (S6). New SPS was used for each sampling and RVA was run. Three replicates were taken from each sample for the following experiments.

### 2.4 Rheological properties

The samples at each stage of gelatinization were immediately transferred to the center of the rheometer testing platform under uncooled conditions. A rotary rheometer (AR-R2, TA instrument Inc., USA) equipped with a 40 mm diameter stainless steel parallel plate fixture at gap 1.00 mm was selected, and silicone oil was applied to the edge of the fixture to prevent water loss. The static rheological characteristics have been tested using a static rheological testing program. The shear rate increased from 0.1 to 300 s^−1^ and then decreased from 300 to 0.1 s^−1^. The shear stress of the sample varied with the shear rate. The measurement of dynamic rheological properties was set with a rheometer temperature of 25 °C and a strain force range of 0.01–100 %. Amplitude scanning is performed at 1 Hz. Frequency sweep tests were conducted to record the storage modulus (G′) and loss modulus (G″) of the slurries. The strain value was chosen as 4 % for all the tests which were obtained by a strain sweep test within the linear viscoelastic region (Xie et al., 2020).

A shear stress of 50 Pa was applied to the samples for 5 min (creep), followed by a shear stress of 0 Pa for 5 min (recovery). The result was expressed as flexibility (Pa^−1^), corresponding to the strain divided by the applied shear stress. Each sample was measured in parallel three times.

### 2.5 Particle size determination

The particle size distribution of the starch samples was determined by a laser diffraction size analyzer (Mastersizer 2000, Malvern Instruments, UK). The starch sample (0.1 g) was mixed with distilled water (5 mL) in a container and then sonicated for 3 min, which dispersed starch particles evenly. The refractive index of starch particles was 1.6, and the refractive index of dispersant was 1.27.

### 2.6 Particle morphology

Scanning electron microscopy (EVO18, ZEISS Co., Ltd., Germany) was used to observe the surface's microstructure of samples. Due to the non-conductive properties of starch samples, it was necessary to coat the sample surface with a conductive layer before scanning. Under vacuum conditions, the microstructure of the sample was observed. SEM images were obtained at 10000× and 500× magnification.

### 2.7 Polarized cross shape observation

Polarized cross observation was performed on the sample using a microscope (BX53M, Olympus Corporation，Japan). 10 mg of the test sample was dispersed into a 1 mL dispersion medium, where the dispersion medium was composed of glycerol and water in a 1:1 volume ratio. The magnification of the microscope was 10,000 and 500 times.

### 2.8 Determination of crystallinity of starch

A wide-angle X-ray diffractometer (XRD-6000×, Shimadzu Instrument Co., Ltd., Japan) was applied to determine the crystalline structure of the starch granules. The test parameters were as follows: Monochromatic Cu-Kα radiation with a wavelength of 0.1542 nm, the tube pressure was 40 kV, the tube flow was 40 mA, the measurement angle was 2θ = 4° ∼ 40°, the step size was 0.0204°, the scanning speed was 6°/min, continuous scanning.

### 2.9 Fourier transform-infrared (FTIR) spectroscopy

The short-range ordered structure of the starch sample was analyzed using an FTIR spectrometer (IRAffinity-1, Shimadzu Instrument Co., Ltd., Japan). The scanning wavenumber range was between 400^−1^ and 4000 cm^−1^ with a resolution of 4 cm^−1^. The spectrum was smoothed, baseline corrected and deconvoluted. The peak intensity at the wavenumber of 1044 cm^−1^ was divided by that at 1015 cm^−1^ to calculate the intensity ratio.

### 2.10 ^13^C nuclear magnetic resonance spectroscopy (^13^C CP/MAS NMR)

The sample was mounted on a 4 mm MAS solid probe and subjected to ^13^C NMR (Magnet 600, Bruker Cooperation, Germany) measurements. The resonant frequency was 150.9 MHz, the rotor rotation rate was 10,000 KHz, the 90° pulse width was 5 μs, the contact time was 1 ms, the delay time was 5 s, and the accumulation times were at least 2400 times. All experiments were done at room temperature (25 °C).

### 2.11 Statistical analysis

The software Jade 6.5 was used to analyze the relative crystallinity of the starch sample. OMNIC software 7.3 was used to analyze the Fourier transform infrared spectroscopy. The ^13^C CP/MAS NMR spectrum of SPS was processed by MestReNova software for peak separation. Analyses of variance by Duncan's test (*p* < 0.05) were conducted using the SPSS 18.0 Statistical Software Program (SPSS Inc. Chicago, IL, USA).

## 3 Results and discussion

### 3.1 Gelatinization characteristics of SPS before and after HMT

Gelatinization is a necessary process for SPS to become sweet potato products. When the temperature reached the gelatinization temperature during starch processing, the starch particles absorbed water and expanded, ruptured, and dissolved in water to form dispersed and continuous starch paste, resulting in an increase in system viscosity. Supplementary material Table 1 showed the gelatinization values of SPS before and after HMT. There was a significant difference(P<0.05) in each parameter between RAW-SPS and HMT-SPS. It is important to note that HMT-SPS had significantly higher peak viscosity (PV), trough viscosity (TV), final viscosity FV and retrogradation value (RV) than RAW-SPS. HMT-SPS under prolonged high temperature and humidity conditions allowed the water to fully penetrate the starch granules to produce the melting phenomenon. This led to a rise in HMT-SPS viscosity. This result conformed with the conclusion of Lei et al. (2020) on maize starch. However, it was found that the viscosity of potato starch decreased after HMT, and further folding between branched and amylose chains after cooling led to a decrease in water absorption, thereby reducing viscosity (Zhao et al., 2020). The increase in gelatinization temperature (GT) after HMT was related to the internal rearrangement of starch particles, indicating the need for more heat for structural decomposition and the formation of paste (Han et al., 2021). The decrease in attenuation value (AV) value after HMT indicated an increase in thermal stability and enhanced resistance to shear of SPS, which was in accordance with the higher PT of HMT-SPS. (Wu et al., 2021) reported that the decreased AV was due to noticeably lower amounts of amylose leached from the restricted swollen starch granules in the HMT starch.

### 3.2 Analysis of the rheological properties

**Fig. 1:**
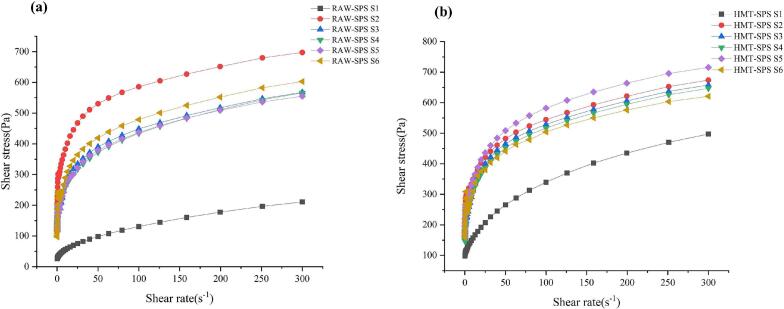
Steady rheological curves of RAW-SPS and HMT-SPS.

The shear stress of RAW-SPS and HMT-SPS increased with the increase of shear rate (Fig. 1). During the S1-S6 stage of gelatinization, the shear stress gradually increased with the increase of shear rate, showing shear thinning. All of the samples presented pseudoplastic behavior, which is similar to the research findings of (Chen et al., 2022). At S1 the shear stress of RAW-SPS and HMT-SPS were lower than that of other stages, at this stage the starch partially gelatinized at 80 °C, with a molecular weight much lower than other stages, and its viscosity was much lower than other stages. At S2, the gelatinization temperature reached 95 °C and the shear stress of RAW-SPS reached its maximum value. However, the maximum shear stress in HMT-SPS occurred at S5. Combined with Supplementary material Table 2, it was found that the lag area increased with the gelatinization process in HMT-SPS. The lag area reflected the differences in starch molecular chains at different gelatinization stages. Both RAW-SPS and HMT-SPS were typical time dependent shear thinning and non-Newtonian pseudoplastic fluids at different stages of gelatinization.

**Fig. 2:**
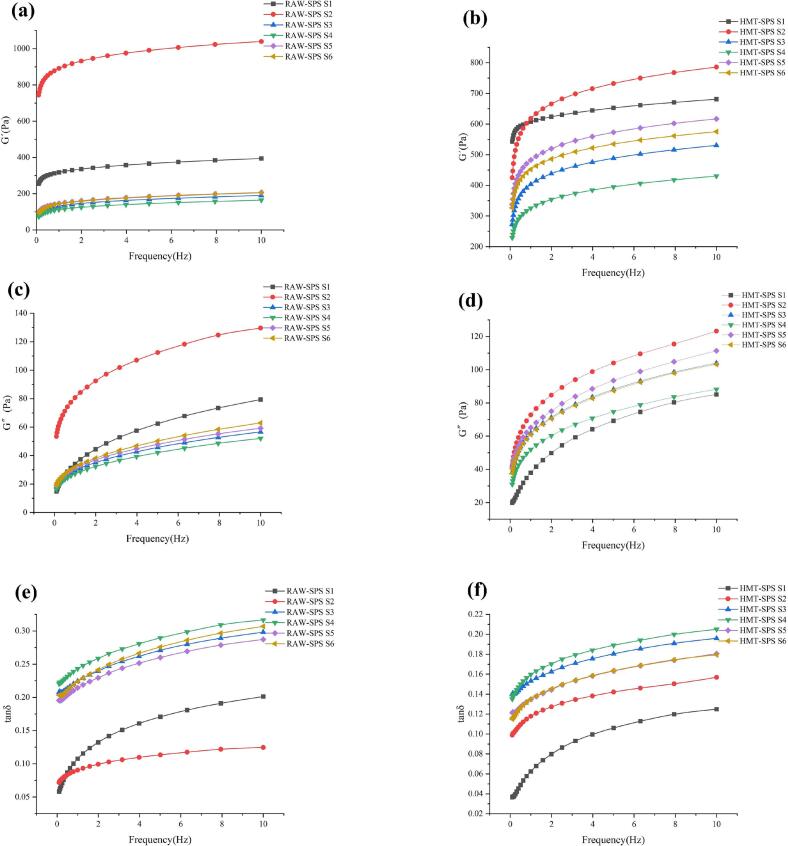
Storage modulus, loss modulus, and tanδ during RAW-SPS and HMT-SPS gelatinization process (a and b are the storage modulus; c and d are the loss modulus; e and f are tanδ.)

The characterization of dynamic rheological properties in food processing is of great significance. The storage modulus(G′) and loss modulus(G″) reflected the elastic and viscous properties of the system, respectively. Tan δ < 1 indicates the predominance of elastic properties of the starch samples (Bora et al., 2023). The dynamic modulus curves of the sample are shown in Fig. 2. G′ and G″ of all starch samples increased with the increase of frequency, indicating that the gel properties of starch were weak. At S2, the modulus (G′ and G″) of RAW-SPS is higher than that of HMT-SPS, which may be due to the reduced swelling ability, and better viscoelasticity. The modulus of HMT-SPS was lower than that of RAW-SPS, and a similar trend has been found in the study of hydroxy propylated SPS (Lee & Yoo, 2011). In the later stage of gelatinization (S3-S6), all samples have decreased, but HMT-SPS was higher than RAW-SPS, indicating that the HMT improved the viscoelasticity of the system, which may be due to the HMT increasing the hydration and swelling of SPS, thereby increasing the number of entangled nodes between SPS molecules and promoting the formation of network structure (Gao et al., 2024). In addition, the hydrophilic groups generated by HMT promoted an increase in the viscoelastic modulus, leading to higher water binding ability (Shi et al., 2022).

From Fig. 2e and f, it was found that the tan δ was less than 0.5, indicating that the samples were weak gel system. The tan δ decreased with increasing frequency, but RAW-SPS had higher the tan δ, indicating that the RAW-SPS had stronger viscosity and fluidity, and had a softer and lower solid content structure. The highest tan δ of RAW-SPS at S4 was due to the softer and more liquid structure of starch gel when it was cooled to 50 °C after full gelatinization. After HMT, the gel structure of starch was more solid. Many studies have found that HMT changed the structure of starch, which changed the structure and rheological behavior of gel, and made samples behave like solids (Ungureanu-Iuga & Mironeasa, 2023).

### 3.3 Analysis of the particle size

The size of starch particles affected starch gelatinization, digestibility, and thermal behavior, which is of great significance in the food industry (Farmakis et al., 2008). According to Supplementary material Table 2, HMT caused changes in the particle size parameters of SPS. Compared with RAW-SPS, the D (0.1), D (0.5), D (0.9), D [4.3], and D [3.2] of HMT-SPS all increased, indicating that the particle size of SPS increased after HMT, which may be due to water molecules entering the interior of starch particles during HMT, causing particle expansion (Olayemi E. Dudu et al., 2019). When the starch particles were large and dissolved slowly, this also resulted in a higher corresponding temperature when the gelatinization was complete, corresponding to the previous gelatinization temperature changes (Yang et al., 2022). Overall, the results indicated that the particle size of SPS increased after treated by HMT. After gelatinization and heating, the starch samples all absorbed water and expanded, resulting in an increase in starch particle size. During the gelatinization process, the particle size showed a trend of first increasing and then decreasing. At S4, both RAW-SPS and HMT-SPS had larger sizes, and when SPS cooled down to 50 °C, starch particles expanded the most and had not yet shrunk, which indicated that starch was still in the expansion stage during the cooling stage of 95–50 °C. Similar kinds of observations are reported for HMT starch (Lin et al., 2019). At each stage of gelatinization, the particle size of HMT-SPS significantly decreased compared to RAW-SPS (*P* < 0.05), which may be due to the sublimation of water during sample preparation and solidification of the expanded volume (Soares et al., 2013).

### 3.4 Analysis of SEM

**Fig. 3:**
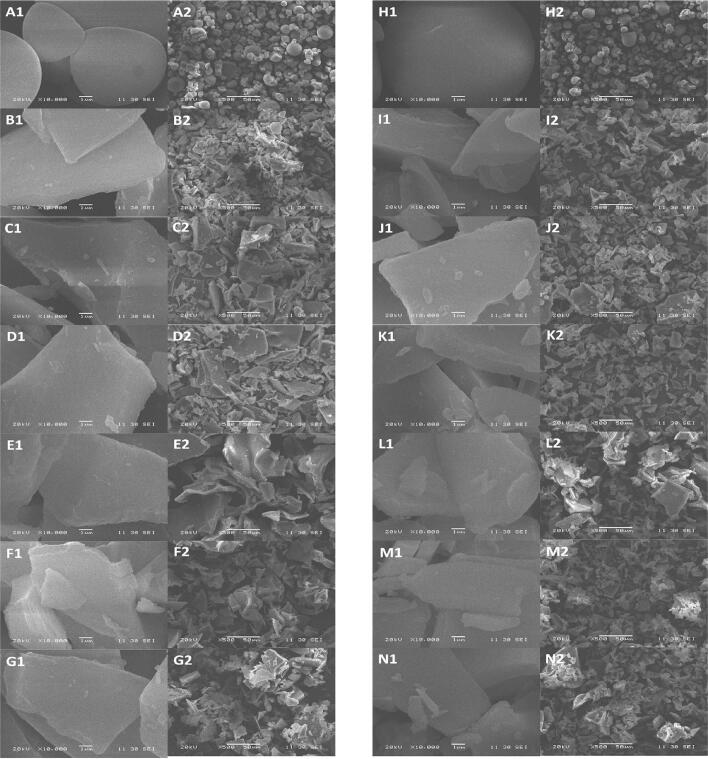
Scanning electron microscopy (SEM) images of RAW-SPS and HMT-SPS (A-G and H–N are RAW-SPS and HMT-SPS, respectively; B-G and I–N indicate six stages of gelatinization. The magnification of images was 10,000× and 500×, represented by numbers 1 and 2 respectively.)

SEM can obtain the three-dimensional morphological structure of the sample, which can comprehensively reflect the structural information of starch particles. Fig. 3 shows SEM images of freeze-dried samples during the gelatinization process using HMT-SPS and RAW-SPS. From Fig. 3A, RAW-SPS exhibited ellipsoidal, polygonal, and irregular shapes when not gelatinized, with a smooth surface without micropores and edges, similar to the images observed by (Zhu et al., 2011). The particle shape of HMT-SPS did not change, but there were slight indentations on the surface, which was consistent with the conclusion of particle size measurement (Sujka & Jamroz, 2013). Fig. 3B and Fig. 3I are an SEM image of gelatinization at 80 °C, during which SPS was in a partially gelatinized state, and a layered structure formed by recrystallization was observed, but no starch particles were found. It was speculated that the remaining starch particles in SPS were encapsulated by soluble starch, resulting in the inability to observe the particle structure. From Fig. 3C–F and Fig. 3J–M, as gelatinization progresses, the layered structure indicated by the particles gradually increases, and amylose precipitates without fully participating in recrystallization. At the same stage, the stripes of HMT-SPS were significantly reduced, thereby, HMT may have reduced the dissolution of amylose in SPS. The study in (Molavi et al., 2018) also found that HMT reduced the leaching rate of amylose.

### 3.5 Polarization microscopy analysis

**Fig. 4:**
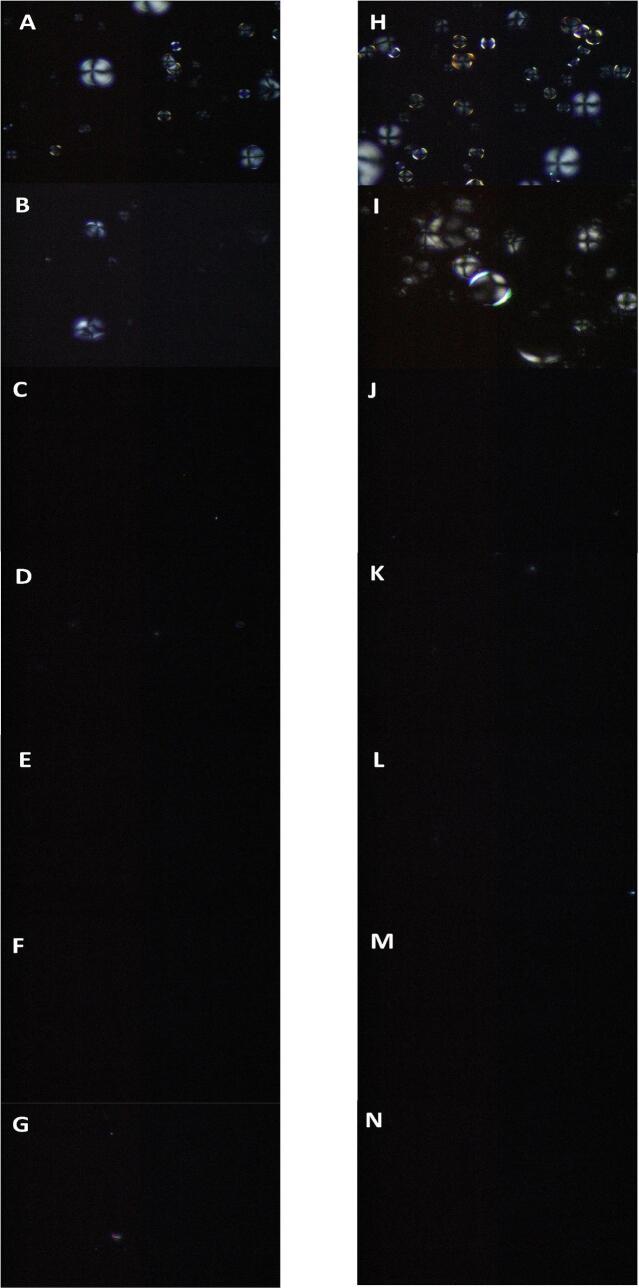
Polarized photographs of SPS and HMT-SPS (A-G and H–N are RAW-SPS and HMT-SPS, respectively; B-G and I–N indicate six stages of gelatinization.)

Starch exhibits birefringence under a polarizing microscope and cross polarized light as a semi crystalline polymer. The intensity of birefringence of starch particles depends on the size of the radius and the helical structure. The polarized light microscopy of samples before and after the HMT was shown in Fig. 4. Compare Fig. 4A and Fig. 4H, the polarization cross of SPS did not significantly weaken before and after HMT treatment, which corresponds to the weak particle size change and basically unchanged particle morphology displayed by HMT. At S1, the polarization cross of HMT-SPS was stronger than that of RAW-SPS. HMT increased the gelatinization temperature from 78.67 °C to 82.55 °C, and the gelatinization degree also decreased, allowing more particle structures to be retained. No polarized cross was found in both groups of S2-S6 stage gelatinization. The reason was that the samples in these stages had been completely gelatinized after being heated at 95 °C, and the original crystalline structure of the starch has been destroyed (Xu et al., 2021). The starch structure formed after recrystallization was significantly different from the original starch particle structure, which was consistent with the particle morphology observed by SEM.

### 3.6 Analysis of starch crystallinity

**Fig. 5:**
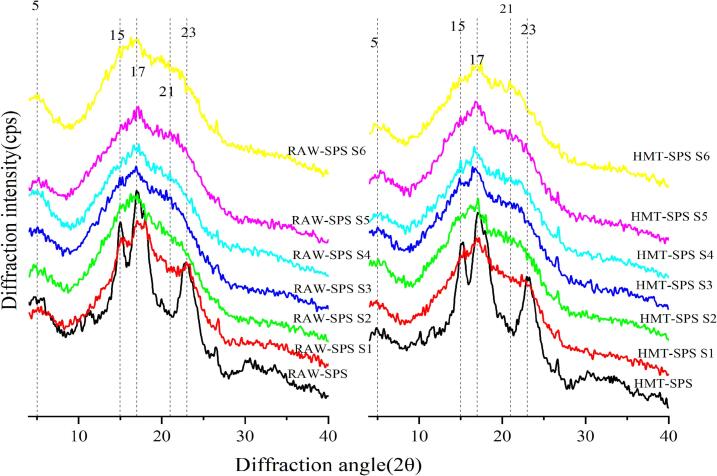
The XRD diffraction curves of RAW-SPS and HMT-SPS at different gelatinization stages(S1-S6).

X-ray diffraction technology is an effective method for studying and measuring the crystalline properties and crystallinity of polycrystalline systems. By comparing and analyzing the position ratio and variation pattern of the peak and dispersion diffraction characteristics in the diffraction curve, the crystalline morphology and properties of starch particles can be determined. Fig. 5 shows the X-ray diffraction curves of RAW-SPS and HMT-SPS at different stages of gelatinization, and the relative crystallinity (RC) is listed in Supplementary material Table 3. RAW-SPS showed absorption peaks near 2θ of 5°, 15°, 17° and 23°, which was a typical C-type starch crystal type (a crystalline mixture of A-type and B-type). The curves also showed a weak peak at 20°, indicating a small amount of amylose-lipid complex. The position of the diffraction peaks of HMT-SPS did not change, indicating that HMT did not lead to a transformation of the crystalline structure of SPS. However, the corresponding intensity of diffraction peaks decreased, which indicated that HMT reduced the strength of starch double helix structure. The RC of SPS increased from 44.97 % to 48.36 % after HMT. It revealed that the high thermal energy during HMT and the migration and movement of water molecules lead to an increase in the degree of ordering and crystallinity of SPS (Zhang et al., 2023). The mechanism of this change can be explained by the fact that the process of HMT promoted the breakage of hydrogen bonds inside the swollen starch particles, the change of double helix, ordered arrangement and tightness of starch molecular chain, and the migration of amorphous regions. Studies by (Ziegler et al., 2018) also found that HMT broke the hydrogen bonds within the oat starch chains or between the starch chains, leading to the displacement of the adjacent double helix structure and the rearrangement of the orientation of the imperfectly parallel arrangement, which ultimately affects the crystalline structure of the starch.

### 3.7 Analysis of the FTIR

**Fig. 6:**
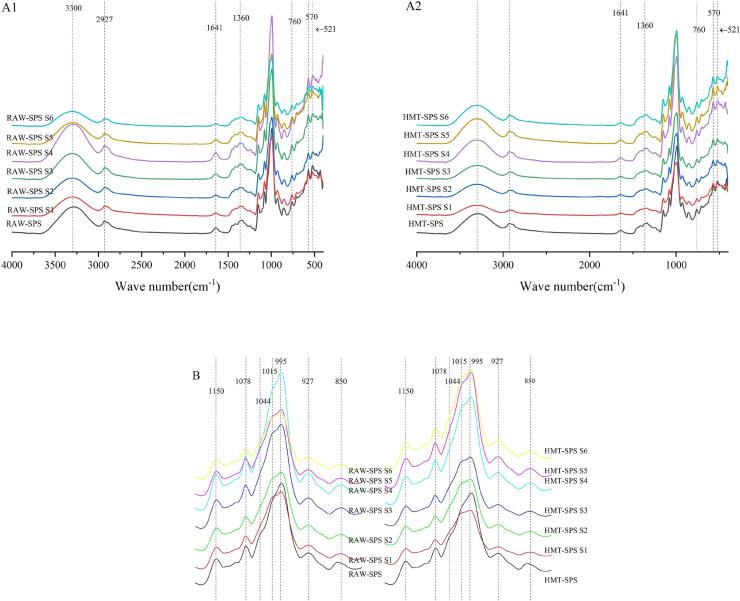
Fourier transform infrared spectra (A1 and A2) and deconvoluted graphs (B) of RAW-SPS and HMT-SPS at different gelatinization stages(S1-S6).

FTIR detect changes in configuration and vibrational frequency of chemical bonds associated with crystals. Infrared spectrum is used to reflect the ratio of ordered structure and amorphous structure in starch. Fig. 6 shows the FTIR spectra of RAW-SPS and HMT-SPS. From the Fig. 6A1 and A2, there were no new absorption peaks generated in each gelatinization stage (S1-S6) before and after HMT, and there was no significant change in the position of characteristic peaks, indicating that HMT and gelatinization process have not changed the starch molecular groups and chemical composition (Zhang et al., 2021). An absorption peak appeared at around 1360 cm^−1^ and 3300 cm^−1^, which was caused by the vibration of C-OH and O-H; An absorption peak appeared at around 2927 cm^−1^, which was a characteristic peak of the starch CH_2_ group; The infrared absorption near 1022 cm^−1^ was a structural feature of the amorphous region of starch, corresponding to the irregular cluster structure of starch macromolecules; The absorption peaks near 850 cm^−1^ and 927 cm^−1^ were the vibrational absorption peaks of the glucose ring. The raw spectra were deconvolved in the 1044–1015 cm^−1^ region to further investigate the effect of HMT on the short-range structure, and the result is shown in Fig. 6B, it can be seen that the characteristic peaks of the crystalline and amorphous regions of the starch sample appeared at 1044 cm^−1^ and 1015 cm^−1^. Therefore, the ratio of 1044 cm^−1^/1015 cm^−1^ can be used to determine changes in the short-range order of the starch system. The higher the ratio of 1044 cm^−1^ and 1015 cm^−1^, the higher the degree of ordering of the starch particles. The characteristic peaks of potato starch also appeared at 1044 cm^−1^ and 1015 cm^−1^ (Li et al., 2016). Based on the results in Supplementary material Table 3, compared with RAW-SPS, the ratio of HMT-SPS increased, which might be due to the repositioning of the double helix structure in the starch crystallization zone or the recombination of hydrogen bonds connecting adjacent double helices and also indicated that HMT was conducive to the short-range ordered crystallization of SPS. However, pre-heating treatment also led to the formation of short starch chains, which reduced the collision possibility of starch chains being in a disordered state due to gelatinization, thereby inhibiting the formation of ordered structures in starch during the retrogradation process.

### 3.8 Analysis of the ^13^C CP/MAS NMR

**Fig. 7:**
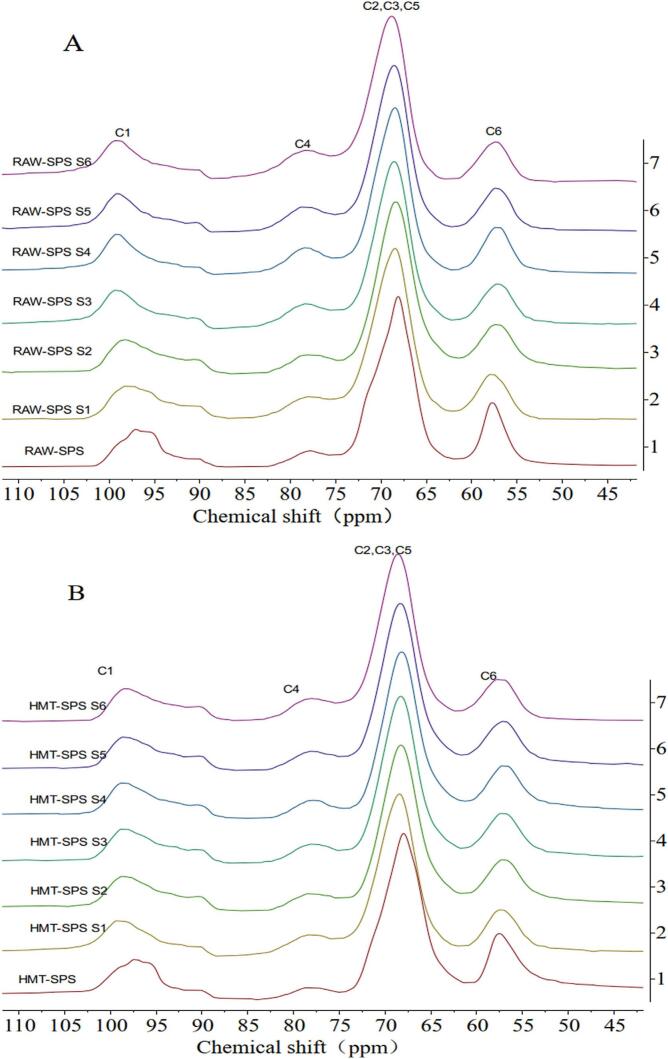
^13^C CP / MAS profiles of RAW-SPS (A) and HMT-SPS (B) at different gelatinization stages (S1-S6).

The helical structure changes of SPS before and after HMT were analyzed using ^13^C CP/MAS NMR, which This further analyzed the short-range structure of SPS. As shown in Fig. 7, SPS generated four main signal intensities before and after HMT and during its gelatinization process, distributed in four regions (C1, C4, C6, and C2/C3/C5) at different chemical shift. HMT did not change the position and shape of these peaks, which indicated that HMT had a slight or no effect on the crystallization type of starch. The ^13^C CP/MAS NMR spectra of SPS were segmented using software, and the chemical shifts of each peak are shown in Supplementary material Table 3. The peak differences of RAW-SPS and HMT-SPS were mainly manifested in the C1 region, where the multiplicity of C1 was related to the crystalline type of starch (Su et al., 2016). RAW-SPS and HTM-SPS both exhibited a quadruple peak in the C1 region, with peaks located at 90.12 ppm and 90.28 ppm respectively belonging to the *V*-shaped starch of RAW-SPS and HMT-SPS. The peaks at 99.19 ppm and 98.97 ppm belonged to the amorphous portion of C1 in SPS. The peaks at 90–98 ppm belonged to the crystalline portion of C1 in RAW-SPS and HMT-SPS, both of which have a triplet peak, which indicated that the SPS before and after HMT were all C-type starch, and there were three un-equivalent residues in the glucose unit of C1 in the sample, with a higher content of A-type crystals and a glycosidic bond configuration β type (Wei et al., 2010). The characteristic peaks of the crystalline portion in the C1 region of the gelatinization stages (S1-S6) of SPS before and after HMT disappeared, but there was no significant change in the characteristic peaks of amorphous and V-shaped starch.

## 4 Conclusion

Exploring the multi-scale evolution of structure during SPS gelatinization under HMT has led to a greater understanding of the impact of HMT during gelatinization process(S1-S6). Modern analytical techniques were used to explore the aggregation mechanism and chain structure of SPS, and the influence of HMT on the multi-scale structure of SPS gelatinization in each stage was discussed. Our previous study certified HMT effectively improved the quality and digestive properties of sweet potato vermicelli. In this study, the SPS after HMT could increase the crystallinity and particle size of SPS, but during the gelatinization process, the particle size was smaller than RAW-SPS. The gelatinization temperature increased to 82.55 °C after HMT. The SPS before and after HMT was mainly C-type starch with A-type crystals. As gelatinization progresses, the characteristic peaks of the crystalline portion in C1 region disappeared, and HMT improved the degree of SPS ordering. The SPS glycosidic bonds before and after HMT were β configuration. The results demonstrated the effects of HMT on the multi angle changes of SPS and could be beneficial for HMT-SPS as a new-style starch to be widely applied in the fields of food and industry. Future research can explore the microstructural changes and changes in the quality of starch-based products in the gelatinization process from a more comprehensive perspective.

## CRediT authorship contribution statement

**Sijie Zhang:** Writing – review & editing, Writing – original draft, Methodology, Investigation, Formal analysis, Conceptualization. **Weiguo Wu:** Methodology, Investigation, Formal analysis. **Jihe Zhu:** Writing – review & editing, Investigation. **Junling Wu:** Investigation. **Zengpeng Gan:** Investigation. **Houqin Deng:** Resources. **Yu Zhang:** Supervision, Resources, Investigation. **Luyan Liao:** Writing – review & editing, Supervision, Resources.

## Declaration of competing interest

The authors declare that they have no known competing financial interests or personal relationships that could have appeared to influence the work reported in this paper.

## Data Availability

Data will be made available on request.

## Appendix A Supplementary data

Supplementary material Image 1
