# Supplementary material for: Multi-scale structural evolution during simulated gelatinization process of sweet potato starch by heat-moisture treatment
Source: Food Chem X. 2024 Dec 24;25:102123. doi: 10.1016/j.fochx.2024.102123 (PMC11741091; doi:10.1016/j.fochx.2024.102123)
Supplement: Supplementary file 1 — Supplementary material [file mmc1.docx]

# Supplementary material

Table 1 Gelatinization characteristic values of RAW-SPS and HMT-SPS.

| Parameters | RAW-SPS | HMT-SPS |
| --- | --- | --- |
| Peak viscosity (PV) (cP) | 3725.33±58.18^a^ | 4149.00±19.31^b^ |
| Trough viscosity (TV) (cP) | 2025.33±43.13^a^ | 3007.00±21.58^b^ |
| Attenuation value (AV) (cP) | 1700.00±16.82^b^ | 1142.00±14.73^a^ |
| Final viscosity (FV) (cP) | 2676.00±50.41^a^ | 4236.33±19.29^b^ |
| Retrogradation value (RV) (cP) | 650.67±15.51^a^ | 1229.33±9.68^b^ |
| Gelatinization temperature (GT) (℃) | 78.67±0.47^a^ | 82.55±0.05^b^ |
| Peak time (PT) (min) | 5.23±0.04^b^ | 5.13±0.00^a^ |

Data were the mean ± standard deviation of at least three replicates. Means with different letters within the same row indicate a significant difference at p < 0.05; RAW-SPS, raw sweet potato starch; HMT-SPS, heat moisture treatment sweet potato starch.

Table 2 Lag area and the particle size of RAW-SPS and HMT-SPS samples.

| Sample | Lag area (Pa/s) | D[4.3] (μm) | D[3.2] (μm) | D(0.1) (μm) | D(0.5) (μm) | D(0.9) (μm) | SSA (m^2^/g) |
| --- | --- | --- | --- | --- | --- | --- | --- |
| RAW-SPS |  | 19.17±0.06^k^ | 13.00±0.00^n^ | 8.08±0.01^d^ | 17.63±0.06^k^ | 32.93±0.12^j^ | 0.46±0.00^a^ |
| RAW-SPS S1 | 2641.80 | 34.30±0.10^h^ | 16.67±0.06^f^ | 6.90±0.01^g^ | 26.70±0.10^f^ | 72.47±0.31^h^ | 0.36±0.00^i^ |
| RAW-SPS S2 | 46006.10 | 50.53±0.12^b^ | 18.87±0.06^e^ | 7.19±0.02^f^ | 34.60±0.10^d^ | 118.67±0.58^b^ | 0.32±0.00^j^ |
| RAW-SPS S3 | 31726.03 | 42.50±0.10^e^ | 19.53±0.06^d^ | 8.16±0.01^c^ | 31.50±0.10^e^ | 93.50±0.26^d^ | 0.31±0.00^k^ |
| RAW-SPS S4 | 29655.41 | 133.33±0.58^a^ | 52.80±0.10^a^ | 27.63±0.06^a^ | 116.67±0.58^a^ | 262.67±0.58^a^ | 0.11±0.00^n^ |
| RAW-SPS S5 | 31388.82 | 45.93±0.40^d^ | 22.53±0.06^b^ | 9.61±0.02^b^ | 37.53±0.15^b^ | 93.33±0.84^d^ | 0.27±0.00^m^ |
| RAW-SPS S6 | 36271.53 | 48.13±0.25^c^ | 19.93±0.06^c^ | 7.84±0.01^e^ | 35.47±0.16^c^ | 107.67±0.58^c^ | 0.30±0.00^l^ |
| HMT-SPS |  | 20.93±0.06^j^ | 13.40±0.00^m^ | 8.18±0.01^c^ | 18.90±0.00^k^ | 36.83±0.06^i^ | 0.45±0.00^b^ |
| HMT-SPS S1 | 7600.42 | 34.40±0.36^h^ | 15.77±0.06^h^ | 6.19±0.01^i^ | 26.53±0.06^f^ | 73.93±0.91^g^ | 0.38±0.00^g^ |
| HMT-SPS S2 | 32081.07 | 32.90±0.00^i^ | 14.60±0.00^l^ | 5.75±0.01^l^ | 24.47±0.06^j^ | 73.03±0.06^gh^ | 0.41±0.00^c^ |
| HMT-SPS S3 | 37870.71 | 35.30±0.46^g^ | 15.67±0.06^i^ | 6.21±0.01^i^ | 26.13±0.16^g^ | 77.77±1.02^f^ | 0.38±0.00^f^ |
| HMT-SPS S4 | 36645.95 | 35.87±0.25^f^ | 16.57±0.06^g^ | 6.77±0.00^h^ | 26.73±0.06^f^ | 78.03±0.57^f^ | 0.36±0.00^h^ |
| HMT-SPS S5 | 40141.37 | 35.23±0.06^g^ | 15.30±0.00^j^ | 6.05±0.00^k^ | 25.67±0.06^h^ | 79.47±0.25^e^ | 0.39±0.00^e^ |
| HMT-SPS S6 | 40830.77 | 32.97±0.15^i^ | 15.10±0.00^k^ | 6.08±0.00^j^ | 25.03±0.06^i^ | 72.67±0.45_h_ | 0.40±0.00^d^ |

D [4.3]: volume-averaged particle size; D [3.2]: surface-area-averaged particle size; SSA: specific surface area; D (0.1), D (0.5), and D (0.9) respectively indicate that 10%, 50%, and 90% of starch particle sizes are less than or equal to this value; Significant differences exist between values with different letters in the same column (p < 0.05); RAW-SPS, raw sweet potato starch; HMT-SPS, heat moisture treatment sweet potato starch.

Table 3 Chemical shifts of ^13^C CP / MAS NMR spectra and short range ordered structural parameters of RAW-SPS and HMT-SPS at different gelatinization stages

| Sample | 1044 cm^-1^/ 1015 cm^-1^ | RC (%) | Chemical shift | | | |
| --- | --- | --- | --- | --- | --- | --- |
|  |  |  | C1 | C4 | C2， C3， C5 | C6 |
| RAW-SPS | 0.616898 | 44.97±2.11^a^ | 99.19， 97.1， 95.13， 90.12 | 77.72 | 71.52， 69.93， 68.1， 66.65 | 57.72 |
| RAW-SPS S1 | 0.607504 | 43.63±1.92^a^ | 97.51， 90.23 | 77.62 | 68.63 | 57.72 |
| RAW-SPS S2 | 0.618215 | 46.05±1.77^a^ | 98.07， 90.45 | 78.88 | 68.40 | 57.22 |
| RAW-SPS S3 | 0.604886 | 42.88±1.23^a^ | 99.07， 90.68 | 78.16 | 68.57 | 57.17 |
| RAW-SPS S4 | 0.568922 | 39.21±1.43^a^ | 99.04， 90.34 | 78.2 | 68.49 | 57.19 |
| RAW-SPS S5 | 0.607627 | 43.91±2.02^b^ | 99.11， 90.31 | 78.18 | 68.58 | 57.32 |
| RAW-SPS S6 | 0.631123 | 50.55±1.73^b^ | 99.04， 90.37 | 78.16 | 68.84 | 57.46 |
| HTM-SPS | 0.627665 | 48.36±1.37^b^ | 98.97， 97.16， 95.3， 90.28 | 78.35 | 70.87， 68.18 | 57.95 |
| HTM-SPS S1 | 0.654562 | 52.31±1.27^b^ | 98.59， 90.48 | 78.27 | 68.54 | 57.25 |
| HTM-SPS S2 | 0.641094 | 51.94±1.44^b^ | 98.03， 90.35 | 77.95 | 68.28 | 57.05 |
| HTM-SPS S3 | 0.632676 | 50.75±1.64^b^ | 98.51， 90.22 | 77.7 | 68.29 | 57.12 |
| HTM-SPS S4 | 0.60391 | 41.92±2.31^b^ | 98.41， 90.44 | 77.75 | 68.21 | 57.98 |
| HTM-SPS S5 | 0.575348 | 39.50±1.94^a^ | 97.91， 90.25 | 77.94 | 68.76 | 57.65 |
| HTM-SPS S6 | 0.59024 | 40.49±1.81^a^ | 98.21， 90.33 | 77.85 | 68.61 | 57.42 |

Significant differences exist between values with different letters in the same column (p < 0.05); RAW-SPS, raw sweet potato starch; HMT-SPS, heat moisture treatment sweet potato starch.
